# Thyroid disorders in alemtuzumab-treated multiple sclerosis patients: a Belgian consensus on diagnosis and management

**DOI:** 10.1007/s13760-018-0883-2

**Published:** 2018-01-25

**Authors:** Brigitte Decallonne, Emmanuel Bartholomé, Valérie Delvaux, Miguel D’haeseleer, Souraya El Sankari, Pierrette Seeldrayers, Bart Van Wijmeersch, Chantal Daumerie

**Affiliations:** 1 Department of Endocrinology, Universitair Ziekenhuis Gasthuisberg, Herestraat 49, 3000 Leuven, Belgium; 2 Department of Neurology, CHU Tivoli, La Louvière, Belgium; 3 Department of Neurology, CHU de Liège, site CHR, Liège, Belgium; 4 Department of Neurology, Universitair Ziekenhuis Brussel, Brussels, Belgium; 5 Nationaal MS Centrum, Melsbroek, Belgium; 6 Department of Neurology, Cliniques Universitaires St Luc, Brussels, Belgium; 7 Department of Neurology, CHU de Charleroi, Charleroi, Belgium; 8 Department of Neurology, Rehabilitation and MS Centre, Overpelt, Belgium; 9 Department of Endocrinology, Cliniques Universitaires St Luc, Brussels, Belgium

**Keywords:** Multiple sclerosis, Alemtuzumab, Autoimmune thyroid disease, Immune reconstitution, Algorithm, Management

## Abstract

This paper deals with thyroid disease that can occur after treatment with alemtuzumab (humanized monoclonal anti-CD52) for relapsing–remitting multiple sclerosis (MS). The 5-year incidence of thyroid adverse events in phase 3 clinical trials is up to 40.7%. In most cases, the thyroid dysfunction is mild and easily manageable and only few serious thyroid adverse events have been reported. The need for patient education on the risk of thyroid dysfunction, as well as regular clinical and biochemical thyroid function screening is well described. However, practical clinical guidance in case of abnormal thyroid-related findings prior to or after alemtuzumab treatment is currently lacking. Therefore, a Belgian taskforce consisting of MS and thyroid experts was created in 2016, with the objective of issuing a clinical thyroid management algorithm based on available scientific evidence and personal experience with regard to alemtuzumab treatment-related thyroid adverse events.

## Introduction

Alemtuzumab is a humanized monoclonal antibody approved for the treatment of adult patients with active multiple sclerosis (MS) in more than 65 countries [1, 2]. In clinical trials, alemtuzumab demonstrated superior efficacy compared to an active comparator (subcutaneous interferon beta-1a) in both treatment-naive patients and in those with inadequate response to prior therapy [3–5]. The mechanism by which alemtuzumab exerts its therapeutic effects in MS is not fully elucidated. However, research suggests immunomodulatory effects through the depletion of mature and circulating B- and T lymphocytes, followed by a repopulation and long-lasting shift in immunological balance, including alterations in the number, proportions, and properties of some lymphocyte subsets, increased representation of regulatory T-lymphocyte subsets, and increased representation of memory T- and B lymphocytes. This mechanism may be relevant to the durable efficacy of this drug [6–8]. Treatment may result in the formation of autoantibodies and increase the risk of autoimmune mediated adverse events. Autoimmune thyroid disease (AITD) has been the most common autoimmune disorder, followed by immune thrombocytopenia (ITP), and nephropathies. According to a hypothesis, differential kinetics of lymphocyte subset reconstitution causing early hyperrepopulation of immature B lymphocytes may explain the occurrence of autoimmune events [9]. A risk management plan (RMP) has been implemented to mitigate the risk of autoimmune conditions in MS patients treated with alemtuzumab. Thyroid function tests, such as serum thyroid stimulating hormone (TSH) levels, should be obtained prior to initiation of treatment and every 3 months thereafter until 48 months following the last infusion. After this period, testing should be performed based on the clinical findings suggestive of thyroid dysfunction. Systematic thyroid function monitoring was integrated as of the phase 2 clinical trial [3]. In the phase 3 clinical trials and the extension study, 40.7% (CARE-MS I) and 37.7% (CARE-MS II) of alemtuzumab-treated subjects developed thyroid adverse events (TAEs) within 5 years, more precisely thyroid dysfunction, with a peak incidence in the third year following the first administration [10–13]. In the event of abnormal findings, the treating physician will have to decide how to manage the sequence of additional tests, possible treatments, and the need to refer the patient to an endocrinologist for further treatment and follow-up. Post-marketing surveillance figures have not been published so far. Due to the current lack of practical clinical guidance with regard to managing thyroid-related autoimmunity after alemtuzumab treatment, a Belgian taskforce was created in 2016 consisting of experts in MS and in thyroid disease, with the objective of issuing practical recommendations to guide the treating physician.

## Methodology

A Belgian taskforce, consisting of 6 experts in MS (E.B., V.D., M.D., S.E., P.S., B.V.) and 2 experts in thyroid disease (B.D., C.D.) first met on May 23, 2016, with the objective of discussing thyroid autoimmunity related to treatment with alemtuzumab based on scientific evidence and personal experience, and designing a clinical management algorithm. During this meeting, the results from the phase 3 CARE-MS I and II and the extension study were discussed along with the major practice points. Next, a draft clinical algorithm was created, which was further developed and discussed through electronic correspondence among the experts, eventually resulting in a consensus clinical algorithm falling into 2 parts: thyroid-related management prior to and post administration of alemtuzumab.

## Autoimmune thyroid disease in the general population

AITD represents the most common autoimmune disease with an estimated prevalence of 5%, and is more frequent in females [14]. The risk of AITD is further increased in the presence of other autoimmune disease(s), such as MS [15]. Usually, AITD presents as 1 of 3 classical phenotypes: autoimmune hypothyroidism or Hashimoto’s disease (HD), autoimmune hyperthyroidism or Graves’ disease (GD), or silent thyroiditis (ST). AITD, however, has to be considered as a dynamic spectrum of diseases, including non-classical phenotypes.

HD is the most common phenotype of AITD. It is a form of painless chronic lymphocytic thyroiditis, and usually slowly develops over years to decades, eventually and most frequently irreversibly leading to hypothyroidism due to thyroid tissue destruction. In most cases, serum thyroperoxidase (TPO) antibodies (Abs) are increased. The treatment consists of lifelong synthetic thyroxine (levothyroxine; LT4) supplementation [16, 17].

GD represents the second most common AITD phenotype, usually developing over weeks to months. GD results in hyperthyroidism, caused by uncontrolled stimulation of the TSH or thyrotropin receptor (TR) by thyrotropin receptor antibodies (TRAbs). TRAbs are thus pathogenic by causing thyroid gland stimulation and thyroid hormone overproduction. Increased TRAb titers represent the hallmark of GD. Rarely, neutral or even blocking TRAbs develop instead of stimulating TRAbs [18]. TPOAbs can also be increased and define the signature of AITD. GD treatment is more complex and consists of reversible medical treatment for 12–18 months with thionamides, anti-thyroid drugs (ATDs) interfering with the TPO enzyme and thus thyroid hormone synthesis, or irreversible non-surgical (radioactive iodine) or surgical (thyroidectomy) treatment [19, 20]. The treatment choice is the result of shared clinical decision between the endocrinologist and the patient. Usually, ATD treatment is well tolerated. However, there are rare cases of severe side-effects, such as agranulocytosis, vasculitis, and liver dysfunction [21]. GD can also target the retro-orbital fat and muscle tissue, resulting in Graves’ orbitopathy (GO), characterized by unilateral or bilateral asymmetrical redness, swelling, diplopia, and very rarely dysthyroid optical neuropathy. The risk of GO is increased with severe hyperthyroidism, high TRAbs, smoking, and in males [22].

ST, the third classical AITD phenotype, is characterized by a self-limiting painless subacute lymphocytic thyroiditis, lasting for weeks to months. TPOAbs are often increased. In the first phase, increased thyroid hormone levels can be observed due to leakage caused by thyroid inflammation, whereas transient hypothyroidism can develop in the second phase. Eventually thyroid function is restored [23].

Thyroid dysfunction and AITD are associated with increased risk for infertility, obstetrical complications, and neuropsychological problems in the offspring [24]. Women with known AITD who are attempting to become pregnant need to be informed of the risks by the endocrinologist and gynecologist. Euthyroidism is mandatory pre-conception. In pregnant women treated with ATDs and/or having persistently high TRAbs, fetal or neonatal hypo- and hyperthyroidism may develop [25].

The diagnosis of AITD is made by measurement of serum thyrotropin or TSH (primary screening parameter for any thyroid dysfunction), thyroid hormones, and antibodies (TPOAbs in case of hypothyroidism, TRAbs in case of hyperthyroidism). If a hyperthyroid patient is symptomatic, nonspecific beta-blockade (propranolol) is indicated independent of the underlying cause. In case of GD and ST, and in case of clinical thyroid gland abnormalities (e.g., goiter), anatomical and functional imaging is indicated by means of ultrasonography and scintigraphy (with radioactive technetium or iodine as tracer), respectively. In a hyperthyroid patient, decreased tracer uptake is suggestive of ST, whereas normal or increased uptake is suggestive of GD [16, 17, 19, 20].

## Autoimmune thyroid disease post-alemtuzumab

AITD is the most frequently observed autoimmune adverse event in MS patients treated with alemtuzumab [3–5, 26]. TAEs can develop from 6 months post-alemtuzumab onward and have a peak incidence in year 3 after the first course and a gradual decline afterwards [10, 11]. In clinical trials, half the patients who tested positive for TPO Ab at baseline and a quarter of patients who tested negative developed a thyroid event. The vast majority (approximately 80%) of patients who presented with a thyroid event after treatment were TPO Ab negative at baseline [27]. In contrast to the general population, AITD after alemtuzumab presents 4 times more often as hyperthyroidism than as hypothyroidism. The thyroid dysfunction is usually mild to moderate, and easily controlled with conventional therapy. Mild cases can resolve spontaneously. Serious TAEs such as GO, neonatal GD, and psychiatric events were rare (1–2%) [28–31]. Furthermore, GD tends to spontaneously resolve in around 1 of 3 cases, which could be due to a higher occurrence of neutral or blocking instead of the classical stimulating TRAbs. Therefore, AITD post-alemtuzumab seems to clinically present as rather ‘non-classical’ phenotypes, also reported in other immune reconstitution circumstances such as HIV treatment, and might, therefore, require a different treatment approach compared to AITD in the general population [32, 33]. Furthermore, the occurrence of thyroid dysfunction did not affect quality of life of alemtuzumab-treated MS patients [34–36].

## Proposal for thyroid-related management prior to the initiation of alemtuzumab

**Fig. 1:**
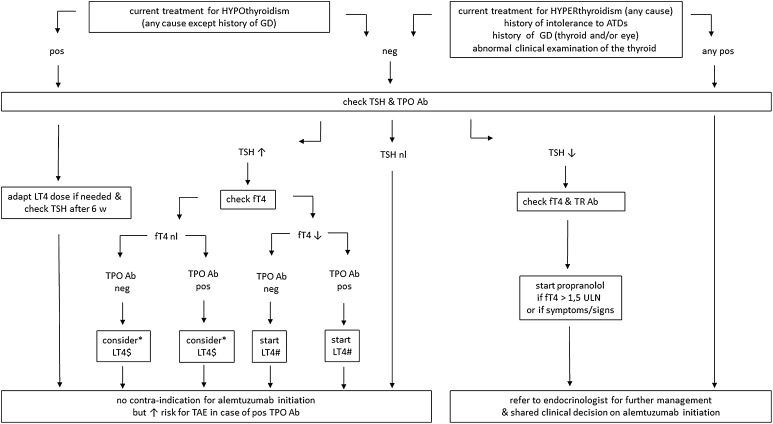
Thyroid management algorithm prior to alemtuzumab. *Ab* antibody, *ATD* anti-thyroid drug, *fT4* free T4, *LT4* levothyroxine, *neg* negative, *nl* normal, *pos* positive, *TAE* thyroid adverse event, *TPO* thyroperoxidase, *TR* thyrotropin receptor, *TSH* thyroid stimulating hormone, *ULN* upper limit of normal. *Symptoms/signs of hypothyroidism or TSH > 10 mU/L favor initiation of LT4; ^$^LT4 ± 0.5 µg/kg/d; ^#^LT4 ± 1 µg/kg/d

Before deciding to treat an eligible patient with alemtuzumab, 2 practical steps should be undertaken with regard to risk assessment for thyroid dysfunction. First, attention should be paid to past or current risk factors known to interfere with thyroid function, including medication and smoking behavior [37]. Second, careful interpretation of the mandatory baseline thyroid tests is needed along with adequate action in case of abnormal findings. The schematical overview of the proposed pre-alemtuzumab clinical management algorithm is shown in Fig. 1.

### Baseline screening

It is important to ask about previous or current problems with thyroid function. In case of current treatment with LT4 for hypothyroidism, measurement of TSH and TPOAb status is indicated to check for the correct supplementation dose and for the nature of the hypothyroidism. In case of current or recent treatment for hyperthyroidism (any cause), current or past GD, known intolerance to ATDs, or the presence of a clinical thyroid abnormality (e.g., a thyroid nodule), the patient should first be referred to an endocrinologist for additional thyroid management, and a shared clinical decision needs to be made with regard to delay of or contraindication for the initiation of alemtuzumab. Finally, ask about intentions with regard to pregnancy desire when dealing with female patients of childbearing age.

### Management in case of abnormal findings

In case of a negative thyroid history and a normal TSH, treatment with alemtuzumab can be started. When a patient is currently treated with LT4 and TSH is abnormal, proper adaptation of the LT4 dose is needed along with a re-evaluation of serum TSH after (minimally) 6 weeks. In the meantime, alemtuzumab can be started without delay.

In case of increased serum TSH at baseline screening, additional free T4 (fT4) testing is indicated. When free T4 levels are decreased, indicative of hypothyroidism, LT4 supplements should be started. When free T4 levels are normal, indicative of ‘subclinical’ hypothyroidism, LT4 treatment can be considered and alemtuzumab can be started.

In case of positive TPOAbs in the patient with or without LT4 supplementation, there is an increased risk for the development of TAEs but alemtuzumab can be started.

When decreased TSH is observed, additional testing of fT4 and TRAbs is indicated, and treatment with propranolol should be started in case of symptoms or signs of hyperthyroidism or a fT4 1.5-fold above the upper limit of normal (ULN). Next, the patient needs to be referred to an endocrinologist for additional thyroid management along with shared clinical decision making on the initiation of alemtuzumab.

## Proposal for thyroid-related management post-alemtuzumab treatment

### Monitoring

**Fig. 2:**
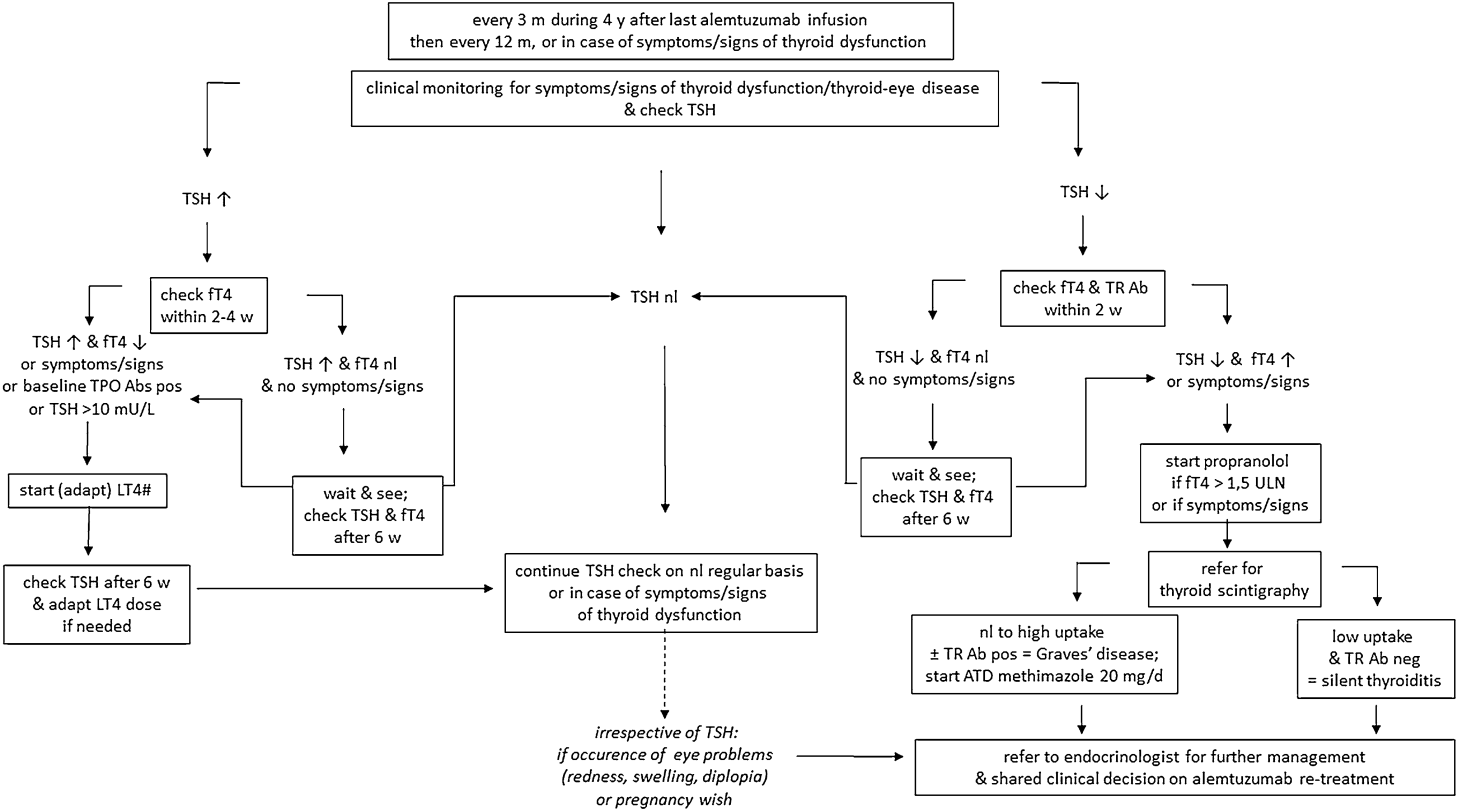
Thyroid management algorithm post-alemtuzumab. *Ab* antibody, *ATD* anti-thyroid drug, *fT4* free T4, *LT4* levothyroxine, *neg* negative, *nl* normal, *pos* positive, *TPO* thyroperoxidase, *TSH* thyroid stimulating hormone, *TR* thyrotropin receptor, *ULN* upper limit of normal. ^#^LT4 ± 1 µg/kg/d, or +25 µg/d in case of dose adaptation

Once alemtuzumab has been administered, clinical screening for symptoms/signs of thyroid dysfunction and thyroid-related eye problems is necessary, along with biochemical monitoring of thyroid function. Education of the patient on symptoms and signs of thyroid dysfunction is warranted. This monitoring is needed at least every 3 months over 4 years after the last alemtuzumab infusion [2]. Beyond that period, the Belgian taskforce proposes to check TSH every 12 months, or in case of symptoms/signs of thyroid dysfunction. Particular attention should be paid to TPOAb-positive patients and to patients with a history of previous AITD, due to their increased risk of a TAE. An overview of the proposed post-alemtuzumab clinical management algorithm is shown in Fig. 2.

### Management in case of abnormal findings

When TSH is normal, one can proceed with regular TSH check every 3 months.

When TSH is increased, fT4 should be checked within 2–4 weeks along with a re-check of TSH. In case of increased TSH but normal fT4, watchful waiting can be adopted with measurement of TSH and fT4 every 6 weeks. In case of spontaneous TSH normalization, a 3-monthly screening of TSH is sufficient. In case of elevated TSH, if fT4 is decreased, or if symptoms/signs of hypothyroidism are present, or if baseline TPOAb status is positive, LT4 is indicated. If TSH is > 10 mU/L, LT4 should be initiated irrespective of the levels of fT4 or antithyroid antibodies. The TSH level should be monitored every 6 weeks for as long as it remains abnormal, and the LT4 dose should be adapted accordingly. When TSH returns to normal under LT4 supplementation, one can proceed with TSH monitoring every 3 months.

If TSH is decreased, fT4 and TRAbs should be checked within 2 weeks along with a re-evaluation of TSH. A watchful waiting approach can be adopted in case of normal fT4 and absence of symptoms/signs of hyperthyroidism, with measurement of TSH and fT4 every 6 weeks. In case of spontaneous evolution towards normal TSH, 3-monthly screening of TSH is sufficient. In case of increased fT4 or symptoms/signs of hyperthyroidism, symptomatic treatment with propranolol should be started in case of hyperthyroid symptoms/signs or fT4 > 1.5-fold the ULN. A thyroid scintigraphy represents the next step in the diagnostic work-up. In case of low tracer uptake and negative TRAbs, the diagnosis of ST is most probable. In case of normal to high tracer uptake and positive TRAbs, the diagnosis of GD can be made, and treatment with an ATD (such as methimazole, thiamazole) can be started at a dose of 20 mg/d per os. In both cases (GD or ST), additional endocrinological advice is indicated with regard to further management.

In the occurrence of uni- or bilateral eye redness or swelling despite a normal TSH (expected to be very rare), the TRAbs should be checked and that patient should be referred to an endocrinologist.

## Pregnancy and thyroid disease in female patients treated with alemtuzumab

Thyroid disease represents particular risks in women who are pregnant. Thyroid dysfunction during pregnancy increases the risk of miscarriage as well as obstetrical and fetal problems. In mothers with GD, maternal TRAbs can be transferred to the fetus, which can potentially result in transient fetal and neonatal hyperthyroidism. Placental transfer and potential pharmacologic activity of alemtuzumab were observed in mice during gestation and following delivery. Women of childbearing potential should use effective contraceptive measures during treatment and for 4 months following a course of alemtuzumab [2].

Additionally, the Belgian taskforce proposes that female patients who want to become pregnant at any time after having been treated with alemtuzumab for MS irrespective of past thyroid function problems, serum TSH should be checked along with TPOAb and TRAb status and a clinical thyroid examination. In case of normal findings there is no contraindication for pregnancy from a thyroid point of view. However, in case one or more abnormal parameters are detected, or if the patient is currently receiving treatment for a thyroid problem, referral is indicated to an endocrinologist for advice with regard to thyroid-related pregnancy risk.

## Conclusions

Available evidence suggests that in case of active MS, potential thyroid-related adverse events usually do not represent a contra-indication for treatment with alemtuzumab, as AITD most often has a mild clinical course. However, autoimmune TAEs occur frequently and necessitate patient education, careful clinical and biochemical monitoring, and adequate diagnostic and therapeutic action if thyroid dysfunction occurs. The proposed clinical management algorithm can serve as a tool in the daily clinical care for the alemtuzumab-treated MS patient. Beyond 4 years after the last alemtuzumab infusion we propose a lifelong yearly measurement of serum TSH as a minimal follow-up. Close interaction and collaboration is needed between the neurologist and endocrinologist, especially in case of (current or past) hyperthyroidism, GO, intolerance to ATDs, and in case of women seeking pregnancy. Finally, there is a need for more insight in the pathophysiology of alemtuzumab-induced thyroid dysfunction. Data on long-term outcomes in the clinical trials will certainly be of great help in the understanding of non-classical post-alemtuzumab AITD.
